# Supplementary material for: Transcription start sites experience a high influx of heritable variants fueled by early development
Source: Nat Commun. 2025 Nov 26;16:10120. doi: 10.1038/s41467-025-66201-0 (PMC12658150; doi:10.1038/s41467-025-66201-0)
Supplement: Supplementary file 1 — Supplementary Information [file 41467_2025_66201_MOESM1_ESM.pdf]

Supplementary Information to
Transcription start sites experience a high influx of heritable variants fueled by early development

Miguel Cortés Guzmán[1,2,*], David Castellano[1,3,*], Claudia Serrano Colome[1,2,*], Vladimir Seplyarskiy[4,5], and Donate Weghorn[1,2,†]

[1]Centre for Genomic Regulation (CRG), The Barcelona Institute of Science and Technology, Dr. Aiguader 88, Barcelona 08003, Spain.
[2]Universitat Pompeu Fabra (UPF), Barcelona, Spain.
[3]Present address: Department of Molecular and Cellular Biology, University of Arizona, Tucson, AZ 85721, USA
[4]Division of Genetics, Brigham and Women's Hospital, Harvard Medical School, Boston, MA, USA.
[5]Department of Biomedical Informatics, Harvard Medical School, Boston, MA, USA.
[*]These authors contributed equally.
[†]Correspondence: dweghorn@crg.eu.

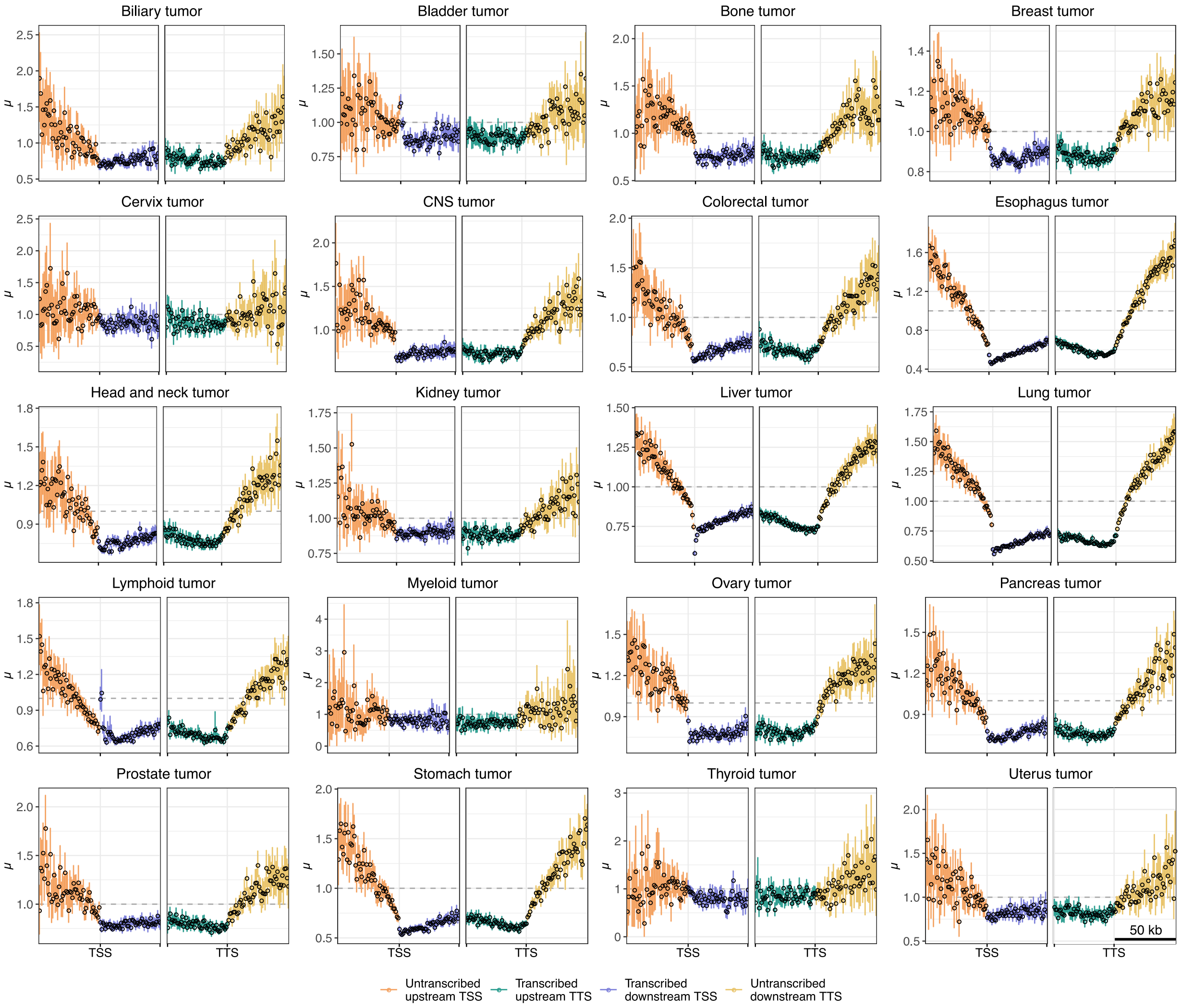

Supplementary Figure 1: Average number of non(CpG>TpG) mutations across 14763 protein-coding genes divided by the expectation based on the 5-mer sequence context, μ, upstream and downstream of the TSS (orange and blue, respectively) and upstream and downstream of the TTS (green and yellow, respectively) in 1-kb windows. Error bars represent the 90% confidence intervals across 100 bootstrap replicates. Results are shown for all used tumour types from the PCAWG dataset, indicated above each plot. Source data are provided as a Source Data file.

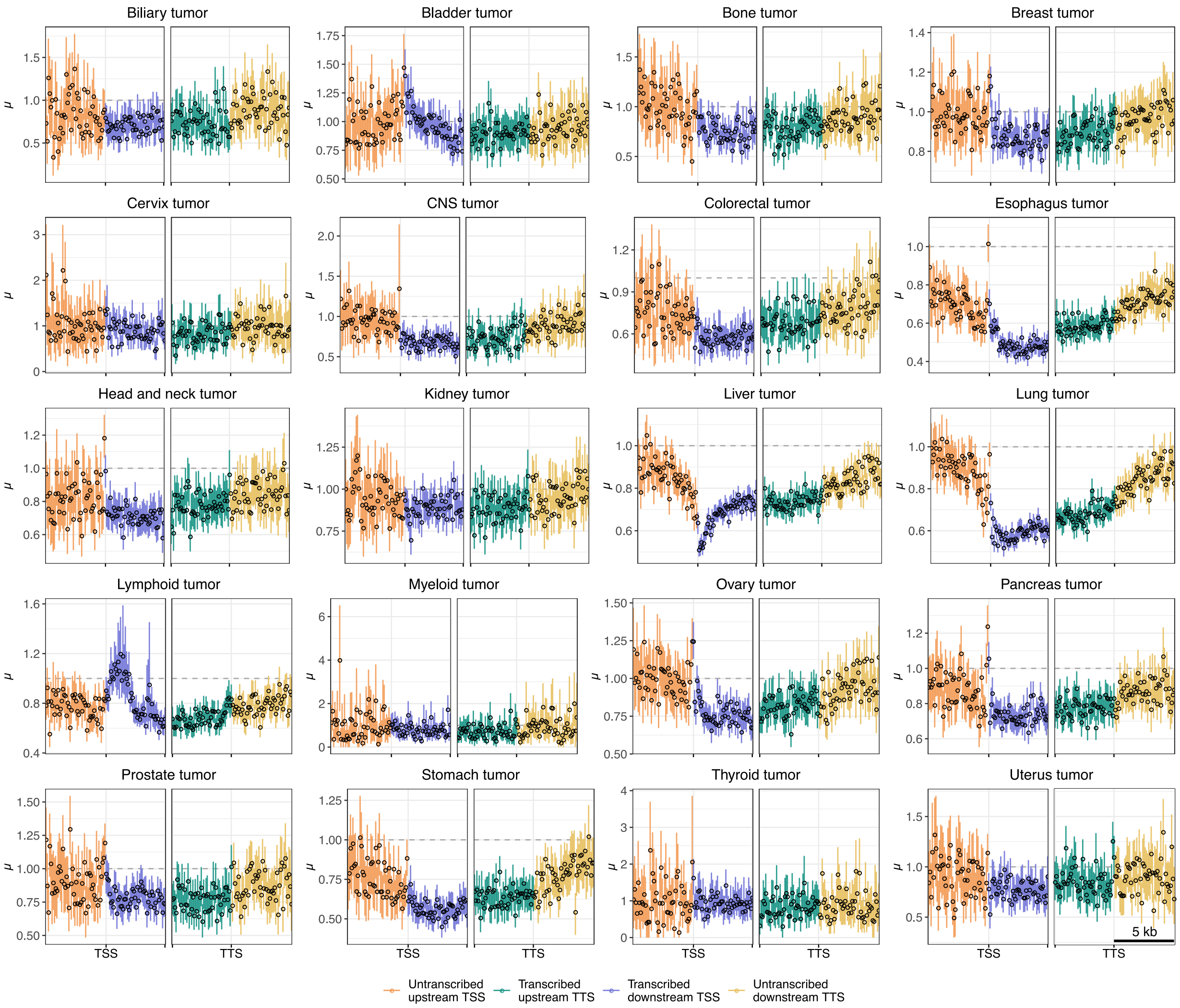

Supplementary Figure 2: Average number of non(CpG>TpG) mutations across 14763 protein-coding genes divided by the expectation based on the 5-mer sequence context, μ, upstream and downstream of the TSS (orange and blue, respectively) and upstream and downstream of the TTS (green and yellow, respectively) in 100 bp windows. Error bars represent the 90% confidence intervals across 100 bootstrap replicates. Results are shown for all used tumour types from the PCAWG dataset, indicated above each plot. Source data are provided as a Source Data file.

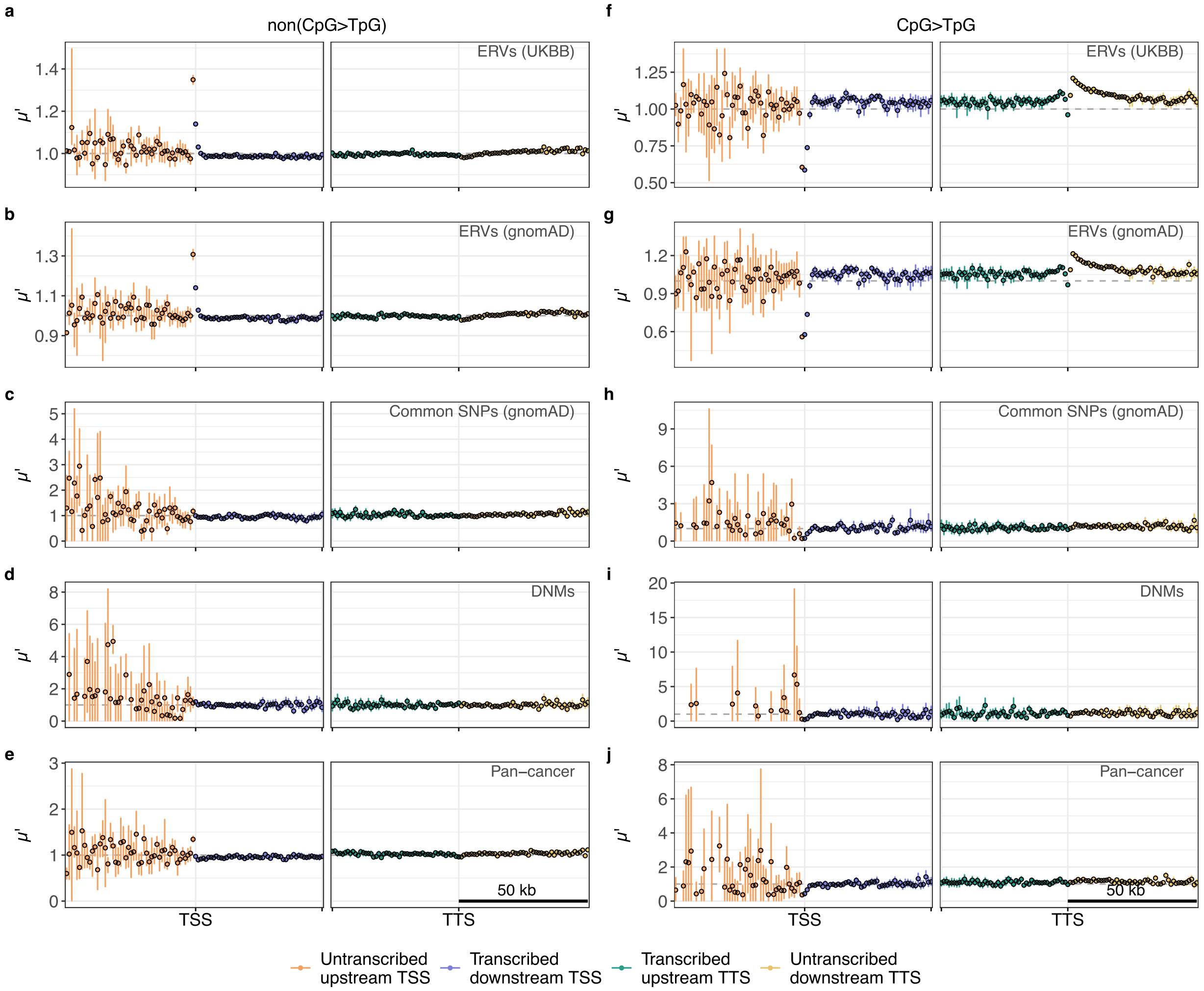

Supplementary Figure 3: Average number of (**a**-**e**) non(CpG>TpG) and (**f**-**j**) CpG>TpG mutations across 3991 divergent long non-coding RNA genes divided by the expectation based on the 5-mer sequence context and by the mean transcript-specific mutation density, μ', upstream and downstream of the TSS (orange and blue, respectively) and upstream and downstream of the TTS (green and yellow, respectively) in 1-kb windows. Error bars represent the 90% confidence intervals across 100 bootstrap replicates. Results are shown for (**a**,**f**) UKBB ERVs, (**b**,**g**) gnomAD ERVs, (**c**,**h**) common gnomAD SNPs (10%<AF<90%), (**d**,**i**) DNMs and (**e**,**j**) PCAWG pan-cancer mutations. Source data are provided as a Source Data file.

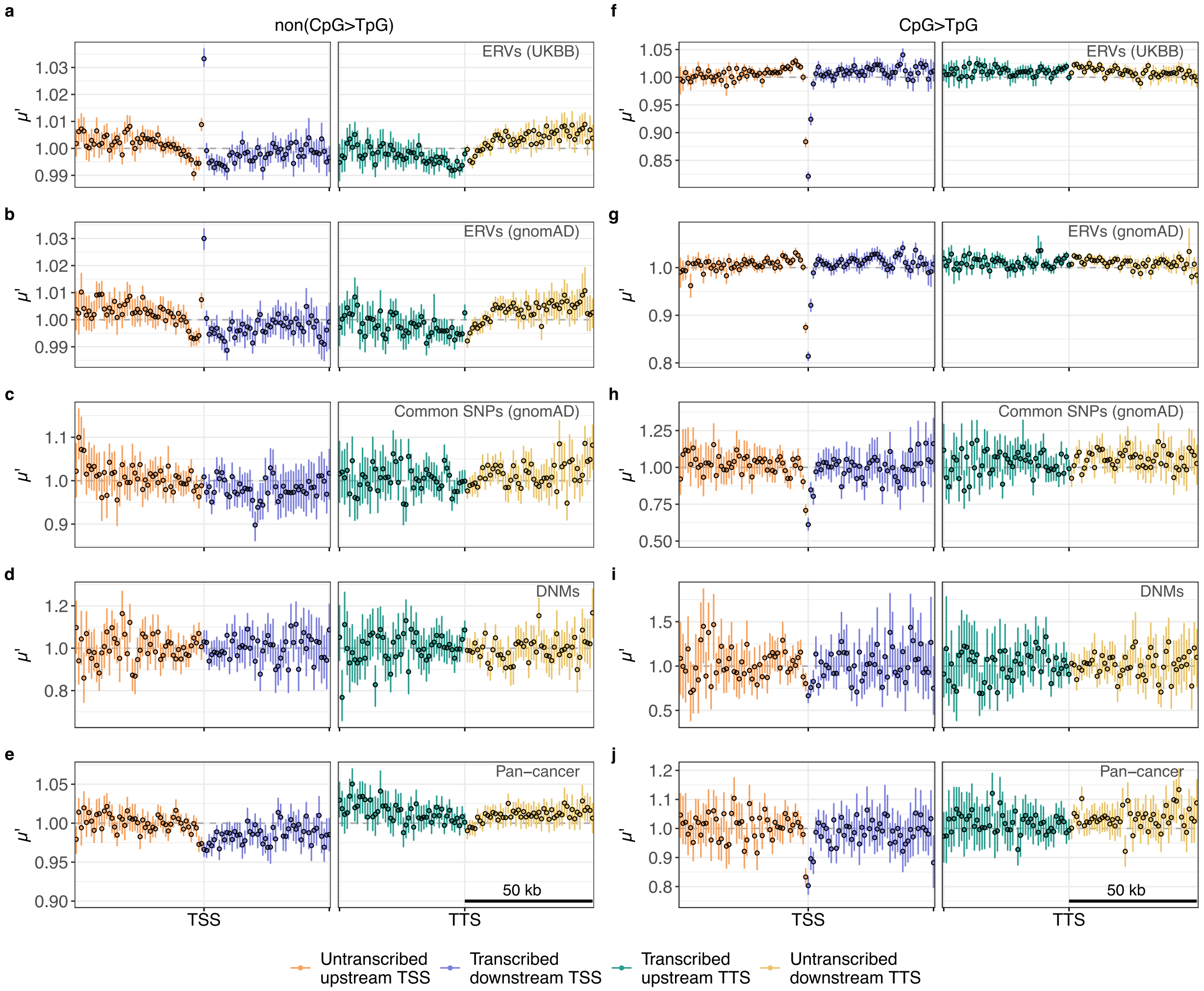

Supplementary Figure 4: Average number of (**a**-**e**) non(CpG>TpG) and (**f**-**j**) CpG>TpG mutations across 8454 intergenic long non-coding RNA genes divided by the expectation based on the 5-mer sequence context and by the mean transcript-specific mutation density, μ', upstream and downstream of the TSS (orange and blue, respectively) and upstream and downstream of the TTS (green and yellow, respectively) in 1-kb windows. Error bars represent the 90% confidence intervals across 100 bootstrap replicates. Results are shown for (**a,f**) UKBB ERVs, (**b,g**) gnomAD ERVs, (**c,h**) common gnomAD SNPs (10%<AF<90%), (**d,i**) DNMs and (**e,j**) PCAWG pan-cancer mutations. Source data are provided as a Source Data file.

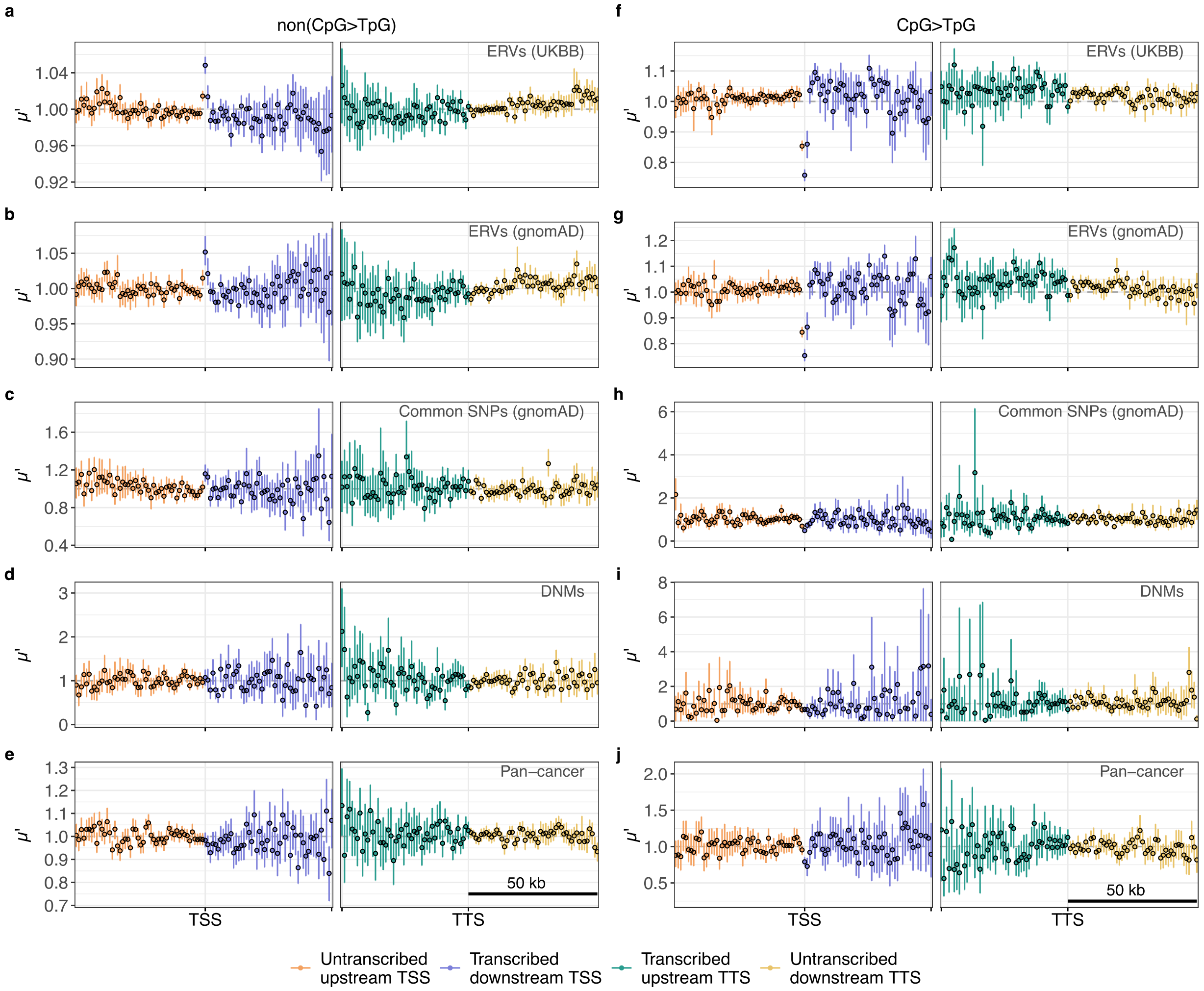

Supplementary Figure 5: Average number of (**a**-**e**) non(CpG>TpG) and (**f**-**j**) CpG>TpG mutations across 1660 pseudogenes divided by the expectation based on the 5-mer sequence context and by the mean transcript-specific mutation density, μ', upstream and downstream of the TSS (orange and blue, respectively) and upstream and downstream of the TTS (green and yellow, respectively) in 1-kb windows. Error bars represent the 90% confidence intervals across 100 bootstrap replicates. Results are shown for (**a**,**f**) UKBB ERVs, (**b**,**g**) gnomAD ERVs, (**c**,**h**) common gnomAD SNPs (10%<AF<90%), (**d**,**i**) DNMs and (**e**,**j**) PCAWG pan-cancer mutations. Source data are provided as a Source Data file.

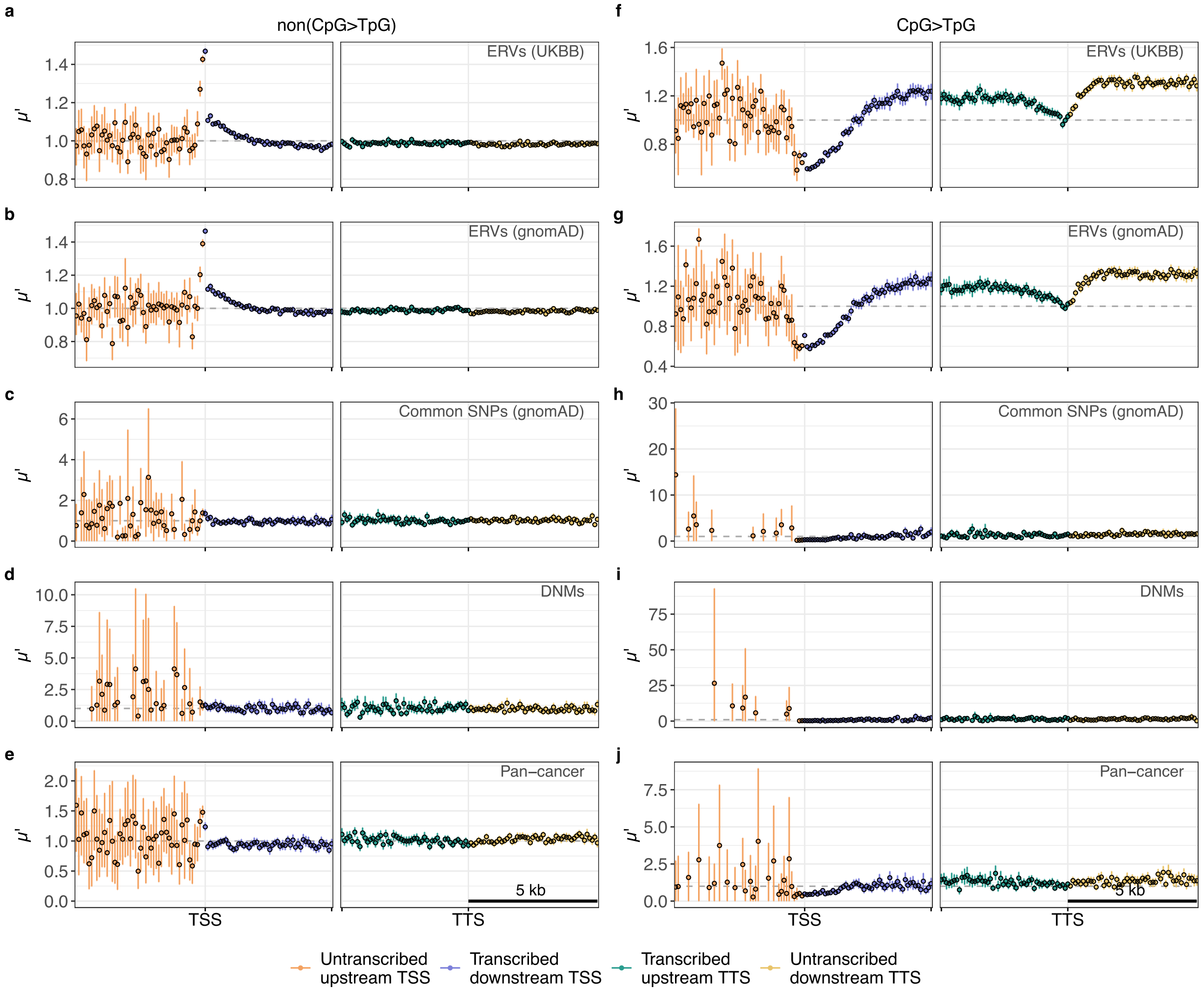

Supplementary Figure 6: Average number of (**a**-**e**) non(CpG>TpG) and (**f**-**j**) CpG>TpG mutations across 3991 divergent long non-coding RNA genes divided by the expectation based on the 5-mer sequence context and by the mean transcript-specific mutation density, μ', upstream and downstream of the TSS (orange and blue, respectively) and upstream and downstream of the TTS (green and yellow, respectively) in 100-bp windows. Error bars represent the 90% confidence intervals across 100 bootstrap replicates. Results are shown for (**a,f**) UKBB ERVs, (**b,g**) gnomAD ERVs, (**c,h**) common gnomAD SNPs (10%<AF<90%), (**d,i**) DNMs and (**e,j**) PCAWG pan-cancer mutations. Source data are provided as a Source Data file.

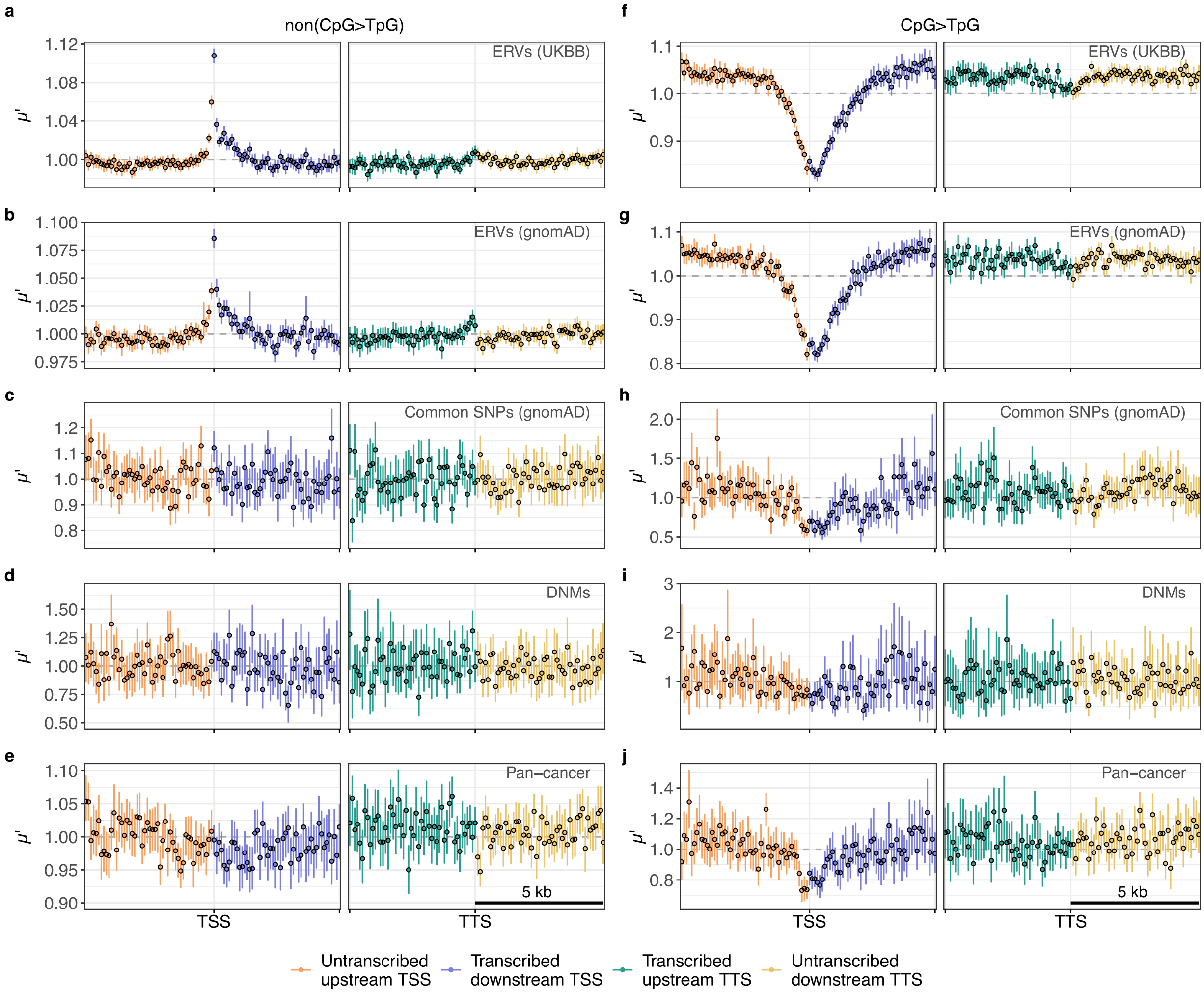

Supplementary Figure 7: Average number of (**a**-**e**) non(CpG>TpG) and (**f**-**j**) CpG>TpG mutations across 8454 intergenic long non-coding RNA genes divided by the expectation based on the 5-mer sequence context and by the mean transcript-specific mutation density, μ', upstream and downstream of the TSS (orange and blue, respectively) and upstream and downstream of the TTS (green and yellow, respectively) in 100-bp windows. Error bars represent the 90% confidence intervals across 100 bootstrap replicates. Results are shown for (**a**,**f**) UKBB ERVs, (**b**,**g**) gnomAD ERVs, (**c**,**h**) common gnomAD SNPs (10%<AF<90%), (**d**,**i**) DNMs and (**e**,**j**) PCAWG pan-cancer mutations. Source data are provided as a Source Data file.

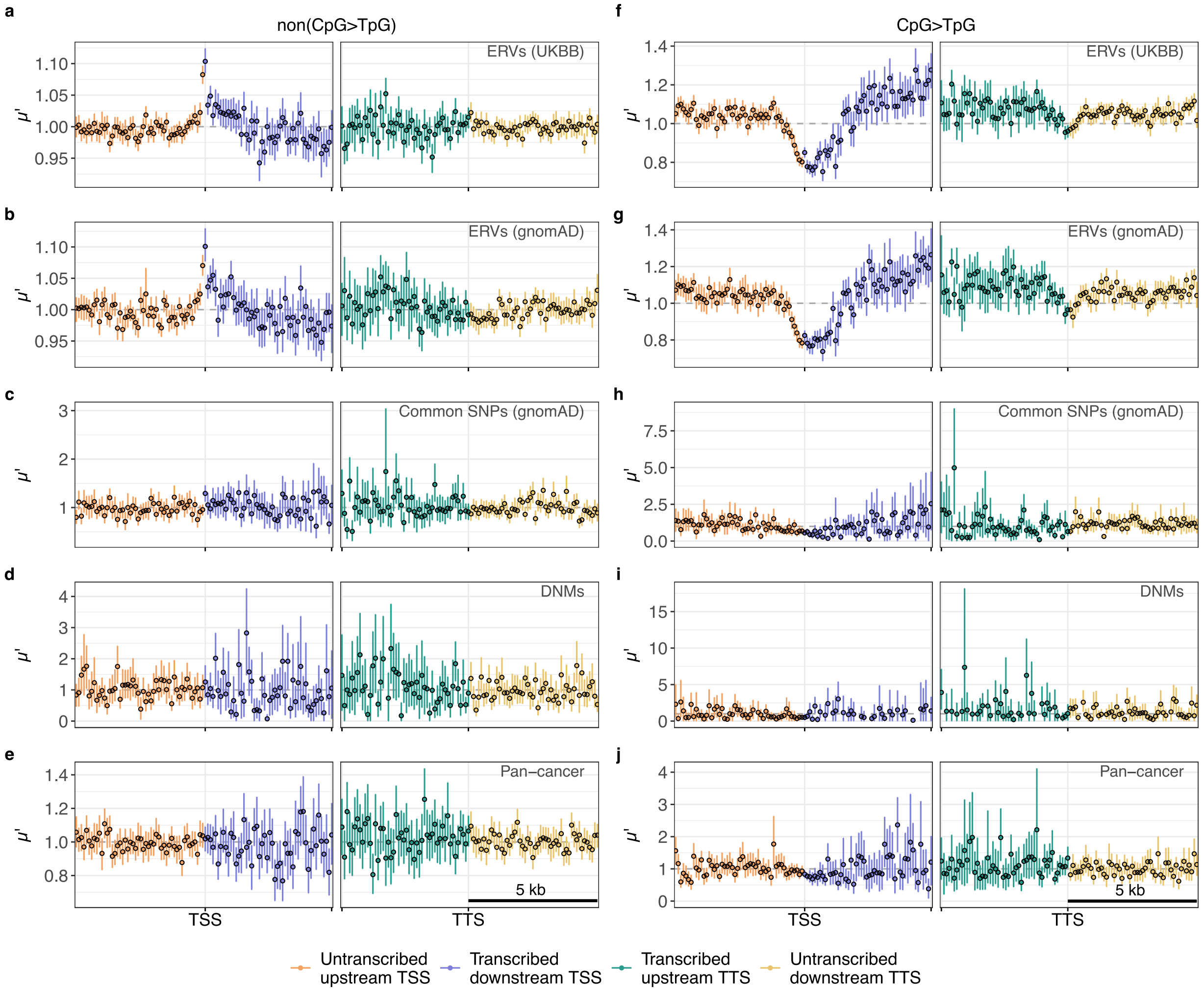

Supplementary Figure 8: Average number of (**a**-**e**) non(CpG>TpG) and (**f**-**j**) CpG>TpG mutations across 1660 pseudogenes divided by the expectation based on the 5-mer sequence context and by the mean transcript-specific mutation density, μ', upstream and downstream of the TSS (orange and blue, respectively) and upstream and downstream of the TTS (green and yellow, respectively) in 100-bp windows. Error bars represent the 90% confidence intervals across 100 bootstrap replicates. Results are shown for (**a**,**f**) UKBB ERVs, (**b**,**g**) gnomAD ERVs, (**c**,**h**) common gnomAD SNPs (10%<AF<90%), (**d**,**i**) DNMs and (**e**,**j**) PCAWG pan-cancer mutations. Source data are provided as a Source Data file.

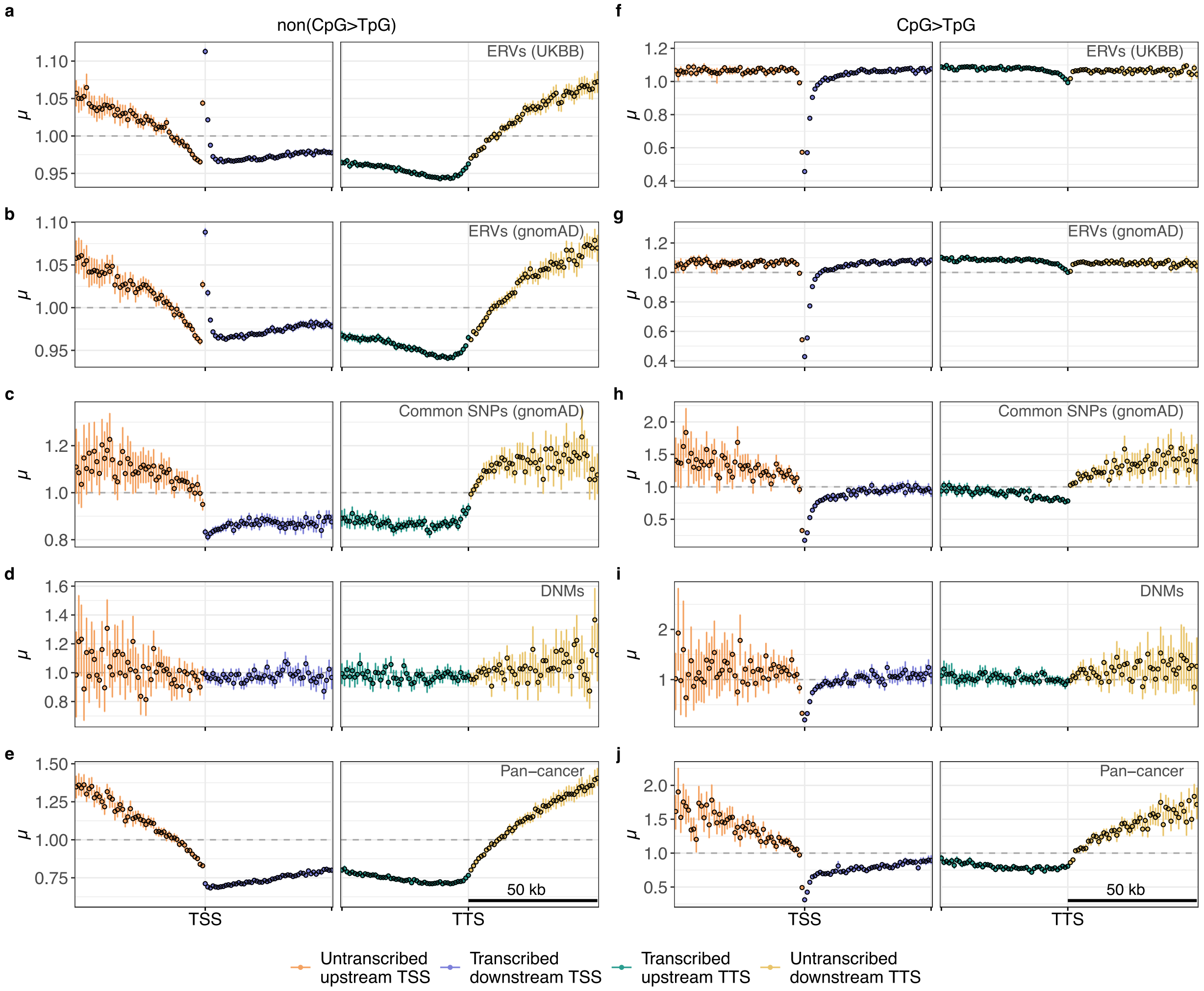

Supplementary Figure 9: Average number of (**a-e**) non(CpG>TpG) and (**f-j**) CpG>TpG mutations across 14763 protein-coding genes divided by the expectation based on the 5-mer sequence context, μ, upstream and downstream of the TSS (orange and blue, respectively) and upstream and downstream of the TTS (green and yellow, respectively) in 1-kb windows. Error bars represent the 90% confidence intervals across 100 bootstrap replicates. Results are shown for (**a,f**) UKBB ERVs, (**b,g**) gnomAD ERVs, (**c,h**) common gnomAD SNPs (10%<AF<90%), (**d,i**) DNMs and (**e,j**) PCAWG pan-cancer mutations. Source data are provided as a Source Data file.

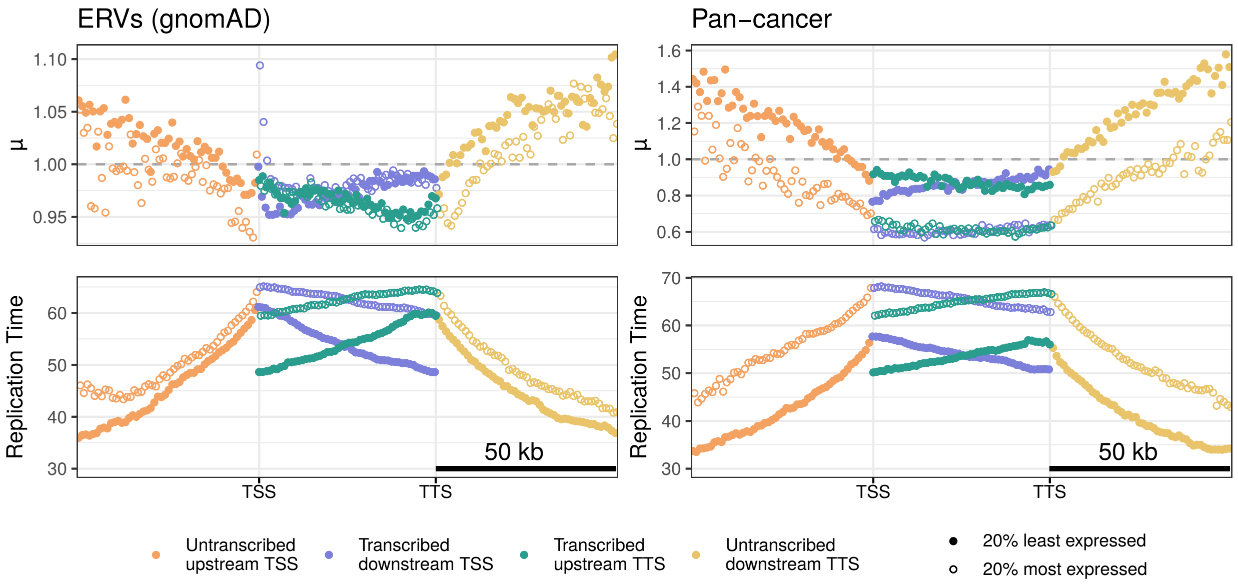

Supplementary Figure 10: μ and average replication time for 1-kb windows of the 20% most highly expressed and the 20% most lowly expressed protein-coding genes. Expression levels from human testis were used for ERVs, while for pan-cancer the weighted mean over tissue-specific expression levels was calculated. The negative correlation between gene length and replication time (note that early corresponds to high and late to low values of the replication time measure) together with the negative correlation between replication time and mutation rate cause the observed positive slope of μ on transcribed regions downstream of the TSS (blue) and a corresponding negative slope on transcribed regions upstream of the TTS (green). Division of each window-specific μ_{tb} by the mean of the transcript (μ_t) produces μ'. Source data are provided as a Source Data file.

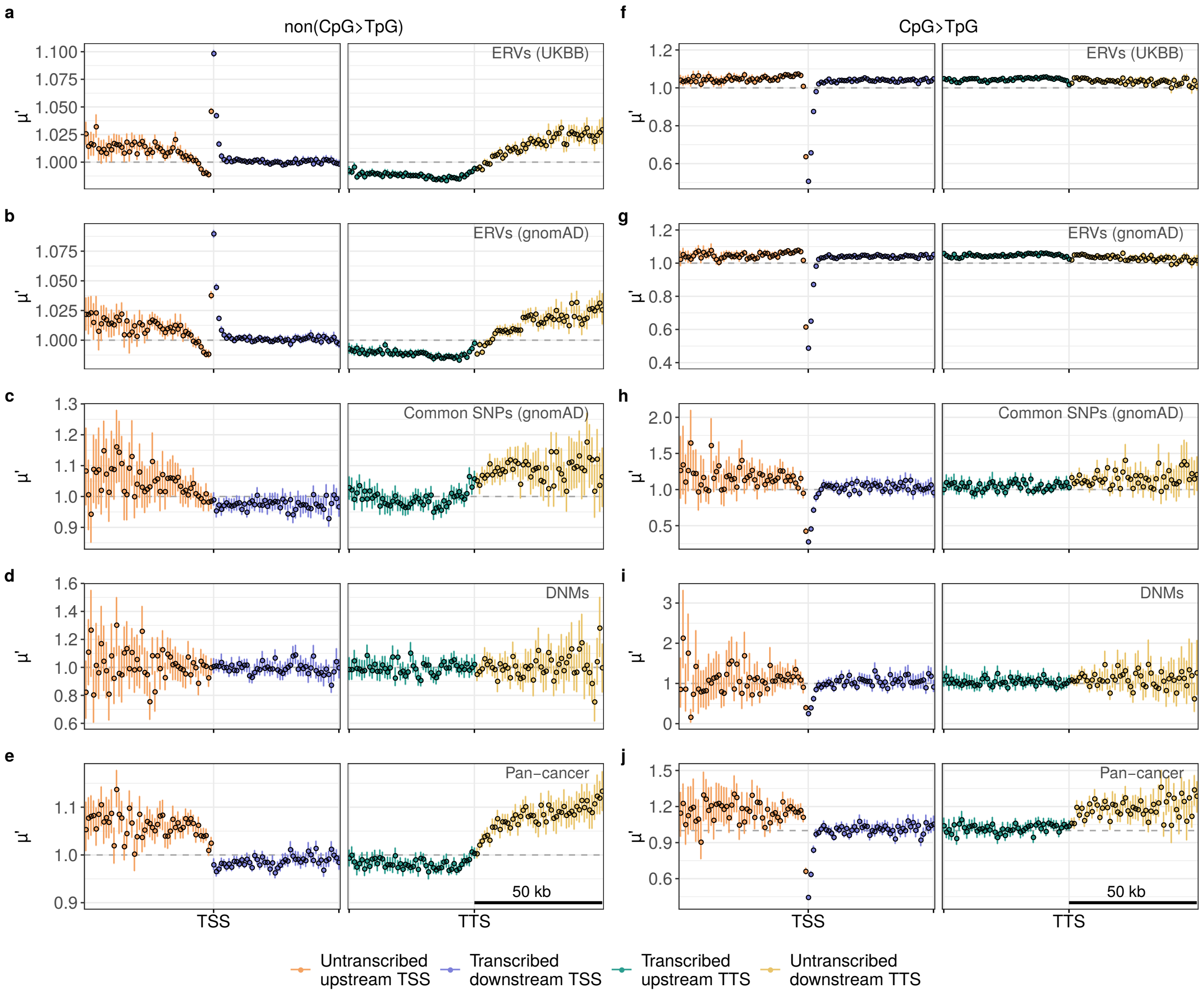

Supplementary Figure 11: Average number of (**a**-**e**) non(CpG>TpG) and (**f**-**j**) CpG>TpG mutations across 14763 protein-coding genes divided by the expectation based on the 5-mer sequence context and by the mean transcript-specific mutation density, μ', upstream and downstream of the TSS (orange and blue, respectively) and upstream and downstream of the TTS (green and yellow, respectively) in 1-kb windows. Coding exons and conserved non-coding sequence elements are excluded. Error bars represent the 90% confidence intervals across 100 bootstrap replicates. Results are shown for (**a**,**f**) UKBB ERVs, (**b**,**g**) gnomAD ERVs, (**c**,**h**) common gnomAD SNPs (10%<AF<90%), (**d**,**i**) DNMs and (**e**,**j**) PCAWG pan-cancer mutations. Source data are provided as a Source Data file.

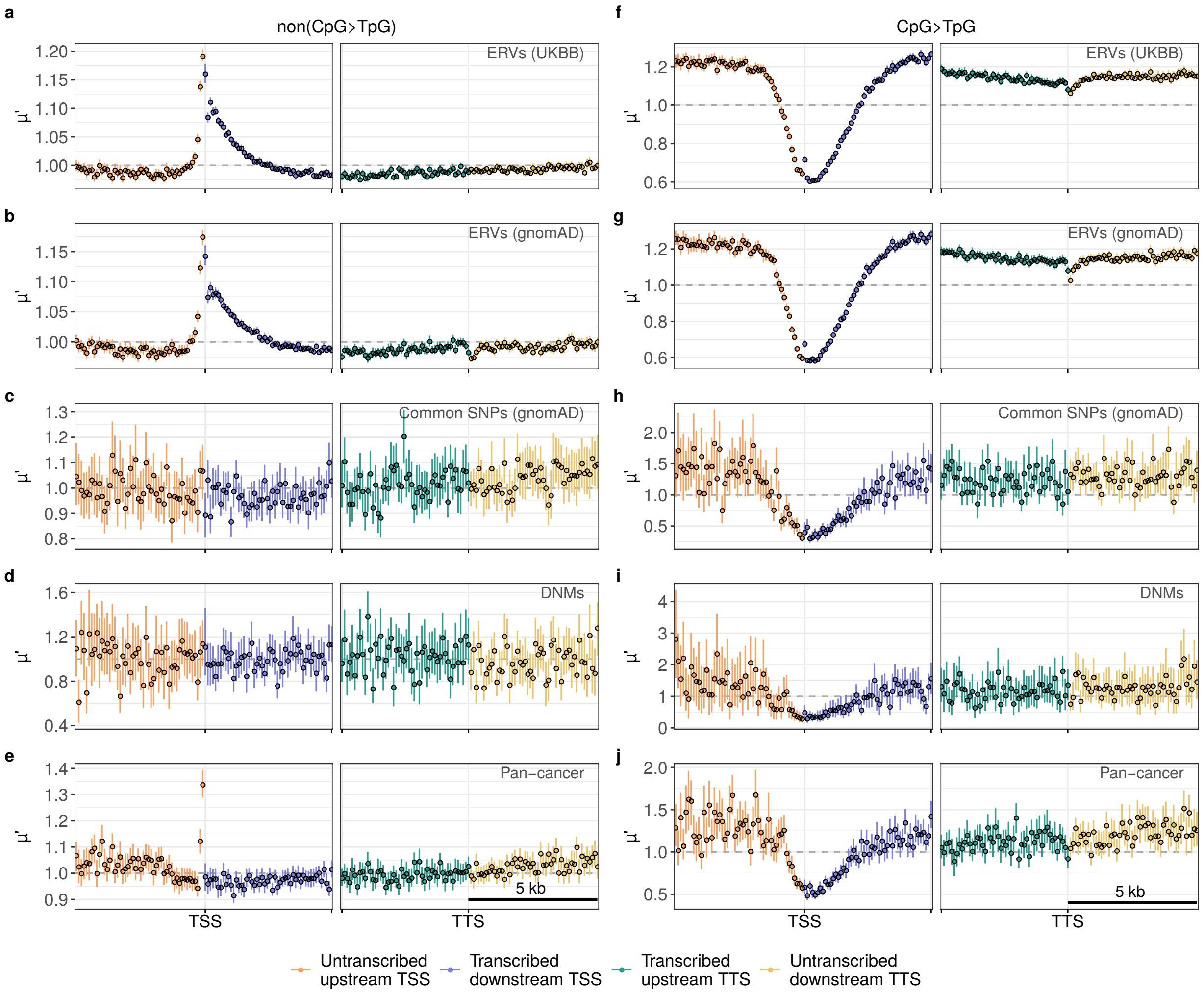

Supplementary Figure 12: Average number of (**a**-**e**) non(CpG>TpG) and (**f**-**j**) CpG>TpG mutations across 14763 protein-coding genes divided by the expectation based on the 5-mer sequence context and by the mean transcript-specific mutation density, μ', upstream and downstream of the TSS (orange and blue, respectively) and upstream and downstream of the TTS (green and yellow, respectively) in 100-bp windows. Coding exons and conserved non-coding sequence elements are excluded. Error bars represent the 90% confidence intervals across 100 bootstrap replicates. Results are shown for (**a**,**f**) UKBB ERVs, (**b**,**g**) gnomAD ERVs, (**c**,**h**) common gnomAD SNPs (10%<AF<90%), (**d**,**i**) DNMs and (**e**,**j**) PCAWG pan-cancer mutations. Source data are provided as a Source Data file.

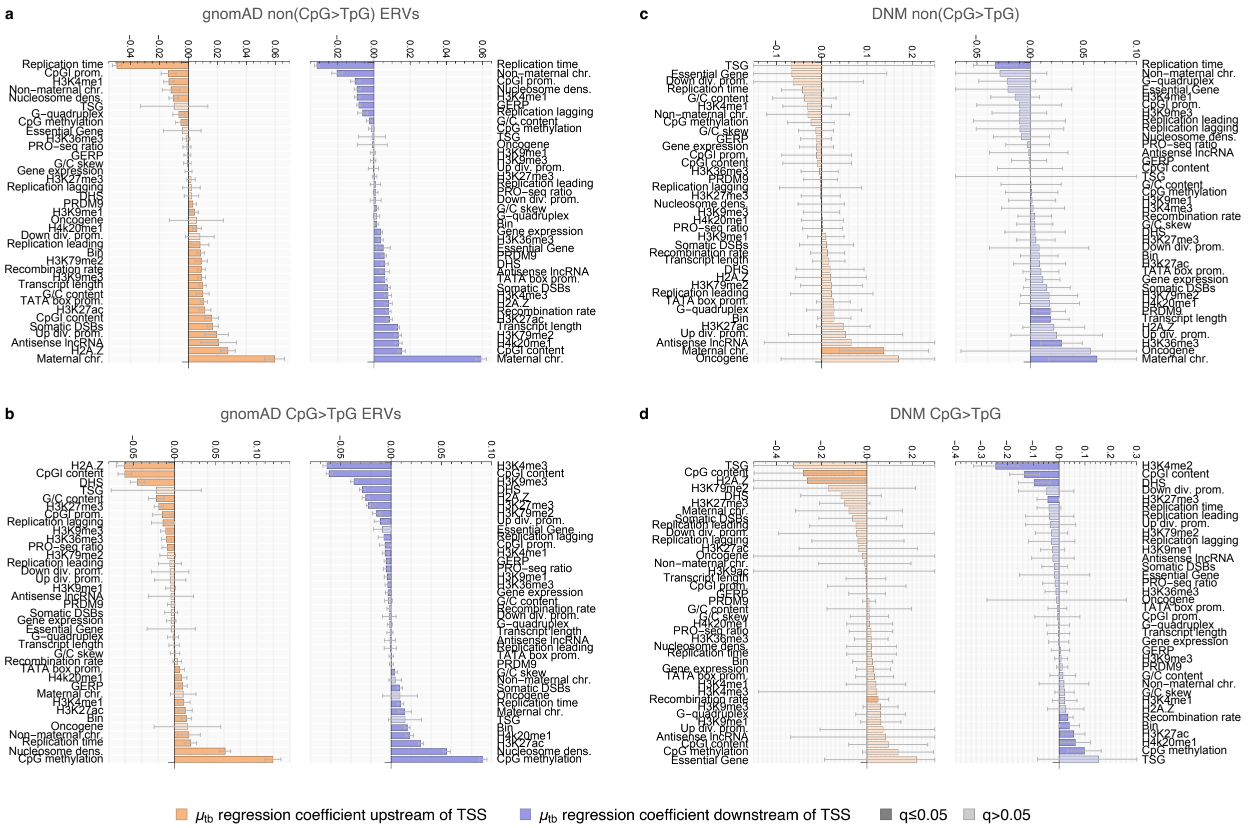

Supplementary Figure 13: Standardised coefficients from multiple negative binomial regression of μ_{tb} across all protein-coding genes and 1-kb windows upstream of the TSS (orange) and downstream of the TSS (blue). **(a)** gnomAD non(CpG>TpG) ERVs, **(b)** gnomAD CpG>TpG ERVs, **(c)** non(CpG>TpG) DNMs, **(d)** CpG>TpG DNMs. Note that early corresponds to high and late to low values of the replication time measure. Error bars show multiple-testing-adjusted standard-error-derived 95% confidence intervals. Dark shading indicates a multiple-testing-adjusted p-value of two-sided z-tests (q-value) of less than 0.05. Correction done with single-step intervals correction.[130] The exact number of windows used for fitting each regression is provided in Supplementary Data 1. Source data are provided as a Source Data file.

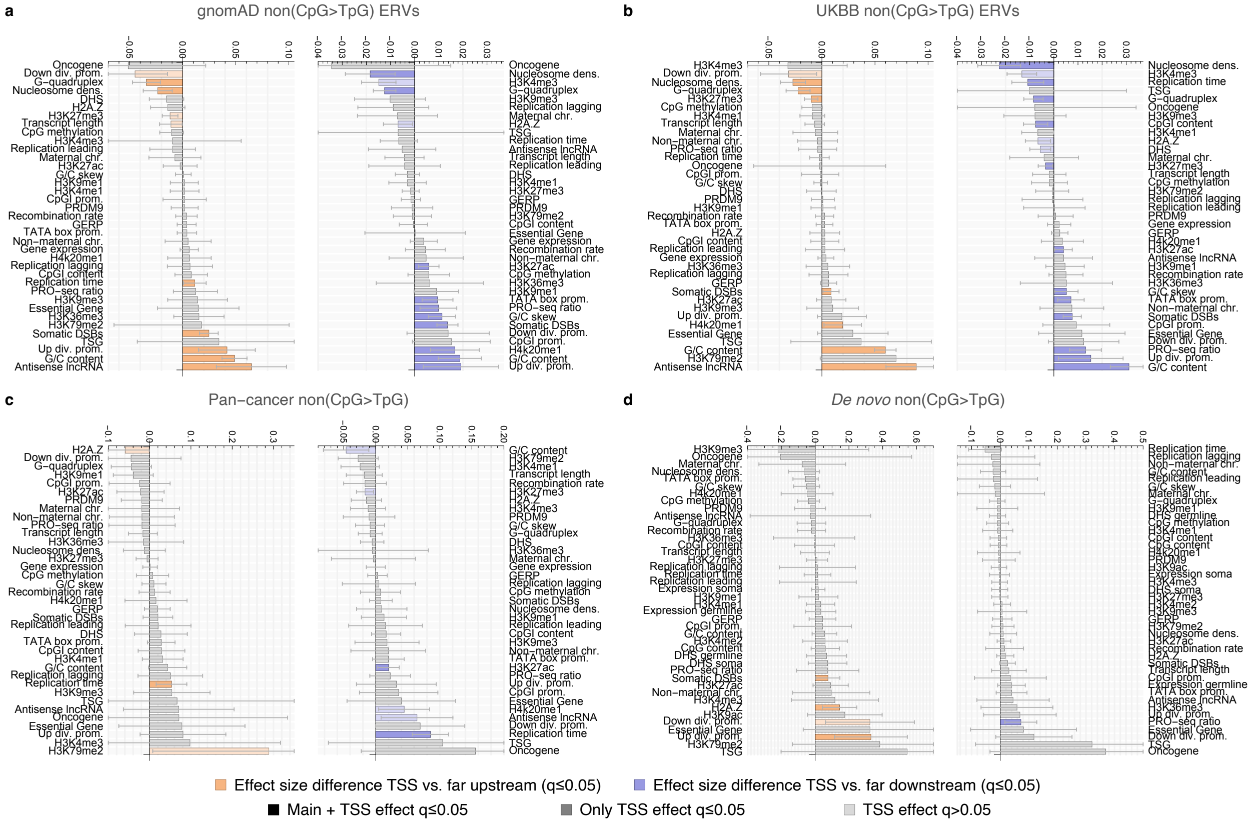

Supplementary Figure 14: (a-c) Difference in standardised multiple negative binomial regression coefficient of regressor×bin interaction between bin brackets [1] and [6,50] kb upstream (orange) and downstream (blue) of the TSS for non(CpG>TpG) gnomAD ERVs (a), UKBB ERVs (b) and pan-cancer variants (c). **(d)** Same as (a), but using single negative binomial regression with interaction terms for *de novo* variants. Regressors with significant TSS interaction effect and significant main effect are shown in dark colors (multiple-testing-adjusted two-sided z-test p-values), those with significant TSS interaction effect but without significant main effect are shown in light colors, and those without significant TSS interaction effect are shown in gray. Error bars represent 95% multiple-testing-adjusted confidence intervals derived from coefficient standard errors. Multiple-testing correction achieved with the single-step intervals correction.[130] The exact number of windows used for fitting each regression is provided in Supplementary Data 2. dens.=density, div. prom.=divergent promoter, chr.=chromosomes, DSBs=double-strand breaks. Source data are provided as a Source Data file.

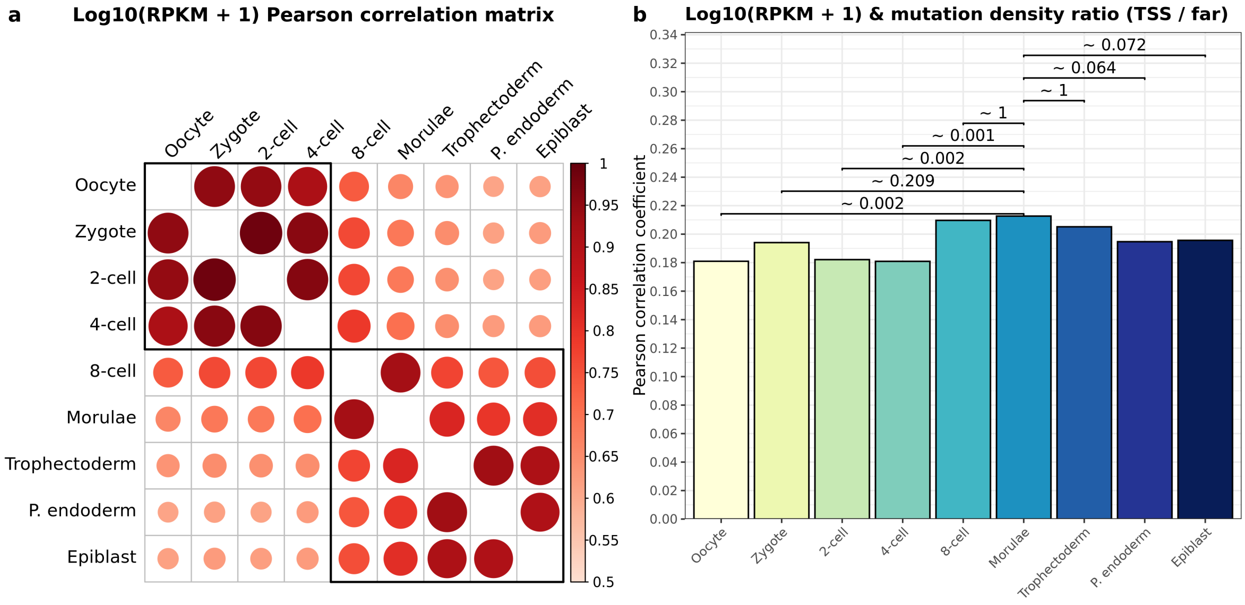

Supplementary Figure 15: (a) Gene expression correlation between chronologically ordered stages of early human development. Two major clusters that can be visually spotted and that consist of transcriptionally similar stages are highlighted. P. endoderm=primitive endoderm. **(b)** Pearson correlation coefficients between gene expression in different early development stages and gnomAD ERVs enrichment at the TSS relative to other analysed regions. For the stage with the highest correlation (Morulae), the multiple-testing-adjusted p-values of pairwise coefficient comparisons are shown in the brackets above the bars (two-sided Hittner tests). All individual correlations in both panels are significant ($q \leq 0.05$, Methods). RPKM=Reads Per Kilobase Per Million. Source data are provided as a Source Data file.

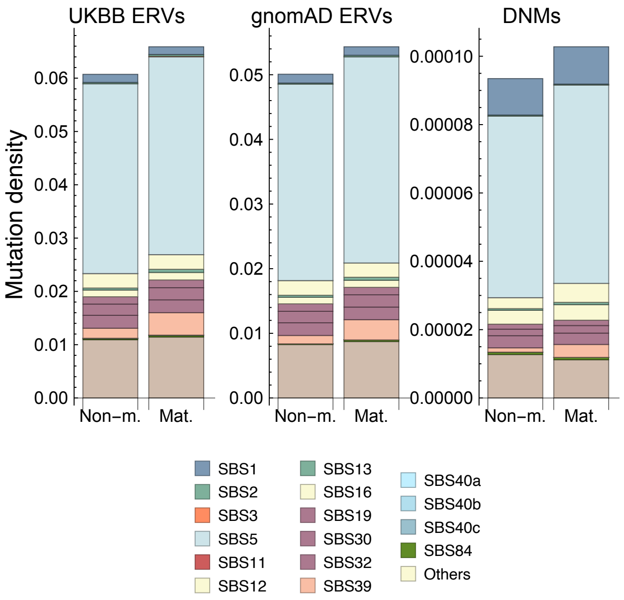

Supplementary Figure 16: Mutation density stratified by mutational signature from all considered genomic regions around genes on non-maternal chromosomes (1, 13, 18, 20) and maternal chromosomes (8, 9, 15, 16) for UKBB ERVs, gnomAD ERVs and DNMs (left to right), showing that SBS39 is increased on maternal chromosomes. Source data are provided as a Source Data file.

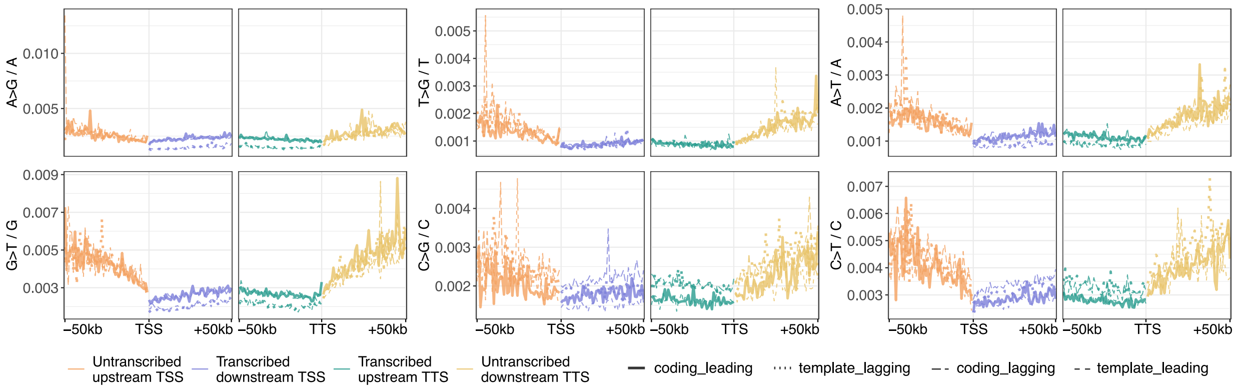

Supplementary Figure 17: non(CpG>TpG) pan-cancer mononucleotide mutation density, stratified by transcription strand (coding or template) and replication strand (leading or lagging) in 1-kb windows around the TSSs of protein-coding genes. Source data are provided as a Source Data file.

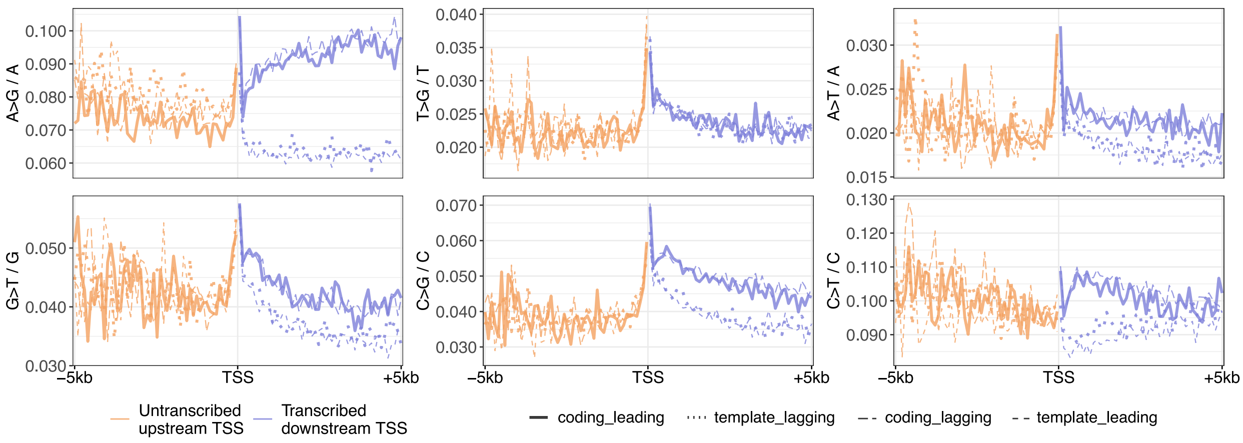

Supplementary Figure 18: non(CpG>TpG) ERV mononucleotide mutation density, stratified by transcription strand (coding or template) and replication strand (leading or lagging) in 100-bp windows around the TSSs of protein-coding genes. Source data are provided as a Source Data file.

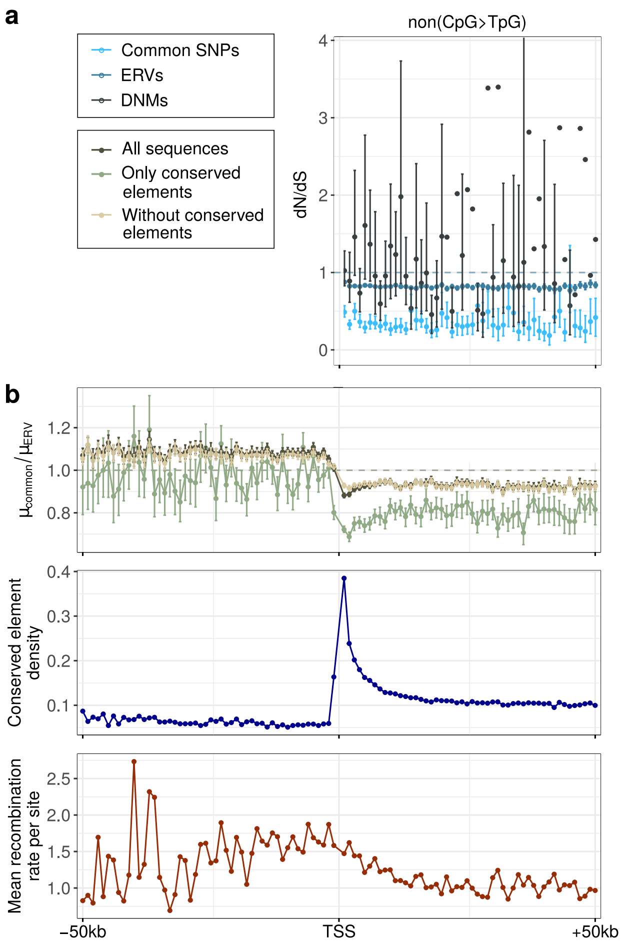

Supplementary Figure 19: (a) dN/dS on exonic regions of protein-coding genes for ERVs, common SNPs and DNMs for non(CpG>TpG), **(b)** Top to bottom: ratio of μ of common SNPs and ERVs for three different filtering criteria (all mappable sequences, only conserved elements and all except conserved elements), density of conserved elements, mean recombination rate per site around the TSS. Note that a slightly elevated value of $\mu_{\text{common}}/\mu_{\text{ERV}}$ on untranscribed sequences upstream of the TSS is likely spurious and due to the genome-wide normalisation of μ_{common}. All shown error bars correspond to 90% confidence intervals derived from 100 bootstraps. Source data are provided as a Source Data file.
