# Supplementary material for: Transcription start sites experience a high influx of heritable variants fueled by early development
Source: Nat Commun. 2025 Nov 26;16:10120. doi: 10.1038/s41467-025-66201-0 (PMC12658150; doi:10.1038/s41467-025-66201-0)
Supplement: Supplementary file 3 — Description of Additional Supplementary Files [file 41467_2025_66201_MOESM3_ESM.pdf]

Description of Additional Supplementary Files

Supplementary Data Legends

Supplementary Data 1: Genome-wide multiple regression results.

Supplementary Data 2: Effect size difference estimates from multiple regression with bin interactions.

Supplementary Data 3: FANTOM5 coordinates and statistical variables of all used genes.

Supplementary Data 4: PCAWG patient identifiers.

Supplementary Data 5: Map between ENCODE cell lines and cancer tissue of origin.

Supplementary Data 6: Map between GTEx tissues and cancer tissue of origin.

Supplementary Data 7: HPO gene set enrichment results for TSS hypermutability signal.

Supplementary Data 8: Gene Ontology (GO) term enrichment results for TSS hypermutability signal.

Supplementary Software Legends

Supplementary Software 1: In-house scripts to calculate the nucleotide-context corrected mutation density across a set of genomic sites assigned to genomic windows based on a set of provided mutations.
